# Evaluation of 18-F-fluoro-2-deoxyglucose (FDG) positron emission tomography/computed tomography (PET/CT) as a staging and monitoring tool for dogs with stage-2 splenic hemangiosarcoma – A pilot study

**DOI:** 10.1371/journal.pone.0172651

**Published:** 2017-02-21

**Authors:** Antonella Borgatti, Amber L. Winter, Kathleen Stuebner, Ruth Scott, Christopher P. Ober, Kari L. Anderson, Daniel A. Feeney, Daniel A. Vallera, Joseph S. Koopmeiners, Jaime F. Modiano, Jerry Froelich

**Affiliations:** 1 Animal Cancer Care and Research (ACCR) Program, College of Veterinary Medicine, University of Minnesota, St. Paul, Minnesota, United States of America; 2 Department of Veterinary Clinical Sciences, College of Veterinary Medicine, University of Minnesota, St. Paul, Minnesota, United States of America; 3 Masonic Cancer Center, University of Minnesota, Minneapolis, Minnesota, United States of America; 4 Clinical Investigation Center, College of Veterinary Medicine, St. Paul, Minnesota, United States of America; 5 Department of Therapeutic Radiology, School of Medicine, University of Minnesota, Minneapolis, Minnesota, United States of America; 6 Division of Biostatistics, School of Public Health, University of Minnesota, Minneapolis, Minnesota, United States of America; 7 Center for Clinical Imaging Research (CCIR) in Diagnostic Radiology, School of Medicine, University of Minnesota, Minneapolis, Minnesota, United States of America; Biomedical Research Foundation, UNITED STATES

## Abstract

Positron Emission Tomography-Computed Tomography (PET-CT) is routinely used for staging and monitoring of human cancer patients and is becoming increasingly available in veterinary medicine. In this study, 18-fluorodeoxyglucose (^18^FDG)-PET-CT was used in dogs with naturally occurring splenic hemangiosarcoma (HSA) to assess its utility as a staging and monitoring modality as compared to standard radiography and ultrasonography. Nine dogs with stage-2 HSA underwent ^18^FDG-PET-CT following splenectomy and prior to commencement of chemotherapy. Routine staging (thoracic radiography and abdominal ultrasonography) was performed prior to ^18^FDG-PET-CT in all dogs. When abnormalities not identified on routine tests were noted on ^18^FDG-PET-CT, owners were given the option to repeat a PET-CT following treatment with eBAT. A PET-CT scan was repeated on Day 21 in three dogs. Abnormalities not observed on conventional staging tools, and most consistent with malignant disease based on location, appearance, and outcome, were detected in two dogs and included a right atrial mass and a hepatic nodule, respectively. These lesions were larger and had higher metabolic activity on the second scans. ^18^FDG-PET-CT has potential to provide important prognostic information and influence treatment recommendations for dogs with stage-2 HSA. Additional studies will be needed to precisely define the value of this imaging tool for staging and therapy monitoring in dogs with this and other cancers.

## Introduction

Positron Emission Tomography-Computed Tomography (PET-CT) is commonly used in human cancer patients to non-invasively diagnose, prognosticate, evaluate therapy-induced changes in tumor metabolism, and monitor response to antineoplastic therapy [1–3]. The clinical application of ^18^F-fluoro-2-deoxyglucose (^18^FDG)-PET-CT as a staging and monitoring tool in veterinary oncology has been limited by the availability and cost of necessary facilities and equipment [4–7]. ^18^FDG is considered the most important radiopharmaceutical for PET in oncology and consists of a glucose analog and the positron-emitting radionuclide ^18^F. Malignant cells rely on anaerobic glycolysis for energy consumption. Anaerobic glycolysis is a relatively inefficient process, resulting in a strongly increased demand for glucose in cancer cells, which can be visualized with FDG. Following administration, FDG extravasates into the interstitium, where it is internalized via facilitative glucose transporters (GLUT), which are present on all cell membranes and especially enhanced on tumor cell membranes. FDG is then phosphorylated into FDG-6-phosphate, which, unlike glucose, is trapped inside the cell and cannot enter the glycolysis pathway [2–8].

A recent study evaluating the effectiveness of this image-guiding method in dogs with various naturally-occurring malignancies showed that ^18^FDG-PET-CT provides information otherwise not obtainable with conventional diagnostic tools, potentially improving the quality of staging and treatment monitoring [7]. This study also suggested that performing PET-CT for staging and therapy control could significantly improve management of dogs with cancer.^7^ Increased uptake of FDG has been demonstrated in canine lymphoma [7,9,10], canine cutaneous mast cell tumors [4] and various solid tumors, including different types of sarcomas and carcinomas, as well as gastrointestinal stromal tumor (GIST) and sertoli cell tumor [6,7]. However, to the authors’ knowledge, this is the first study to evaluate the utility of ^18^FDG-PET-CT as a staging and monitoring tool in dogs with hemangiosarcoma (HSA).

HSA is a malignant neoplasm of blood vessel forming cells. It is highly metastatic and invariably fatal in dogs, resembling human angiosarcoma. It occurs primarily in the spleen, followed by right atrium, skin and subcutis, and liver [11]. Metastases can be widely spread, affecting lungs, liver, omentum, mesentery, skeletal muscles, kidneys, brain, and bones. The rate of concurrent right atrial masses in dogs with splenic HSA varies between 8.7% (2 out of 23 dogs) [12] and 25% (6 out of 25 dogs) [13]. It has been reported that a right atrial mass may be visible via echocardiography in 65–90% of the cases [14–17]; however, this might be an overestimate since blood clots within the pericardium can produce a mass effect. Thoracic radiography is generally recommended to screen for pulmonary metastatic disease with a reported 78% sensitivity and 74% negative predictive value for detecting pulmonary manifestations of HSA while abdominal ultrasonography is the recommended staging tool for abdominal lesions. The reported sensitivity and negative predictive value of thoracic radiography for cardiac HSA are 47% and 43%, respectively [18]. The prognosis for dogs with splenic HSA remains extremely poor with less than 10% of cases surviving to 12 months [19]. Studies performed in the last decade using new combinations of old drugs [20], different treatment modalities [21], and small molecule tyrosine kinase inhibitors [22–23] have shown no benefit over standard of care surgery and doxorubicin chemotherapy. We previously showed that canine HSA tumor-initiating cells express epidermal growth factor receptors (EGFR) and urokinase plasminogen activator receptors (uPAR), and that these cells are effectively killed by a bispecific EGF-urokinase angiotoxin (eBAT) designed to target EGFR and uPAR simultaneously [24]. We then extended these studies to examine feasibility, safety, and efficacy of eBAT in dogs with naïve HSA, in the minimal residual disease setting (post-splenectomy) [25]. Because the absence of lesions on conventional staging tools does not exclude HSA, there has been increasing interest in finding more accurate staging modalities that can detect early metastatic and/or locally recurrent disease. For this reason, owners of dogs enrolled in the eBAT clinical study, called SRCBST-1 [25], were given the option to pursue a PET-CT for staging of their pet’s cancer in addition to routine radiography and ultrasonography to determine its utility in identifying potential metastatic lesions and/or disease progression. Improved staging could have a profound impact on prognostication of disease and predictability of possible complications once treatment is instituted. Likewise, improved sensitivity of diagnostic tools could greatly improve monitoring of tumor response and influence management of cancer patients. Data from mRNA sequencing studies show high expression of GLUT1, GLUT3, and vascular endothelial growth factor receptors (VEGFR1 and VEGFR2) in all canine HSA samples analyzed, supporting the likelihood that these tumors avidly take up glucose and thus the use of ^18^FDG-PET-CT to diagnose systemic disease [26]. We thus designed this study to evaluate the ability of ^18^FDG-PET-CT to detect potential cancerous lesions in dogs with stage-2 splenic HSA following splenectomy, as compared to conventional radiography and ultrasonography. We hypothesized that ^18^FDG-PET-CT would allow for identification of potentially malignant lesions that were otherwise undetectable by routine imaging modalities.

## Materials and methods

### Dog population, eligibility, and ethics statement

Dogs that received PET-CT scans were undergoing diagnosis and treatment for HSA at the Veterinary Medical Center (VMC), University of Minnesota (UMN) according to a clinical trial investigating safety and efficacy of eBAT administered following surgery (splenectomy) and prior to five cycles of doxorubicin chemotherapy starting on Day 21 [25].

All imaging studies were conducted with approval and under the oversight of the UMN Institutional Animal Care and Use Committee (IACUC Protocols 1110A06186 and 1507-32804A). Inclusion criteria for PET-CT scanning included: histopathologic diagnosis of splenic HSA obtained following splenectomy; no evidence of gross regional metastasis at surgery, nor regional or distant metastasis from standard diagnostic modalities (thoracic radiography and abdominal ultrasonography); acceptable risk profile for general anesthesia; normal blood glucose level at baseline; and written informed client consent. There were no restrictions based on age, gender, neuter status, or other physical parameters. If abnormalities were noted on the PET-CT that were not visualized on routine staging tests, owners were given the option for their dogs to have a subsequent PET-CT on Day 21 after completion of the experimental eBAT therapy and prior to initiation of doxorubicin.

### PET-CT scan procedure

PET-CT scans were performed at the Center for Clinical Imaging Research (CCIR), UMN, after splenectomy and before chemotherapy administration. Dogs were fasted for a minimum of 12 hours with water freely available. Pre-medication with diazepam (Hospira, Inc. Lake Forest, IL 60045) at 0.1 mg/kg and butorphanol (Zoetis, Inc. Kalamazoo, MI 49007) at 0.2 mg/kg was used to minimize anxiety and muscular activity during the radiopharmaceutical uptake period [27]. Blood glucose was measured via glucometer prior to intravenous injection of ^18^FDG, with 60-min uptake time prior to PET-CT scanning. Dogs were anesthetized using propofol (Bayer HealthCare LLC, Shawnee Mission, KS 66201) for induction (5 mg/kg or to effect) and isoflurane/oxygen mixture via endotracheal tube for maintenance, placed in sternal recumbency, and whole-body CT and PET data (100% of body coverage) were acquired with a standard protocol using Siemens mCt-64 Biograph TrueD HD PET/CT scanner fitted with high resolution crystals (HD PET) (Minneapolis, MN PET matrix 256 x 256, fwhm 5, zoom 1, 3 iterations with 12 subsets, filter = Gaussian, slice thickness 1.0 mm). High resolution, diagnostic quality CT was employed for all studies. The scanner was cross calibrated between the dose calibrator and scanner and all clocks were synchronized. The protocol started with pre and post contrast CT images acquired from head to tail using CareDose4D and a kernel of B30f medium smooth with a slice thickness of 1.0 mm and 0.8 mm slice interval. The whole-body emission PET scan of the same region followed at 3 min per bed table position at a resolution of 256 x 256. Images were subsequently reconstructed into 3 mm slice thickness by 3mm slice interval images, CT matrix 512 x 512, slice thickness 1.0 mm, fov = 600 mm. The reconstruction method used was TRUEX (HD-PET) with a gaussian filter with ordered-subset expectation maximization algorithm. Attenuation correction of PET images was performed using the CT data. Contrast medium (Omnipaque 240^™^, GE Healthcare, Minneapolis, MN; Iohexol 240 mg/ml) was given at a dose of 2.0 ml/kg.

### Image analysis

All images were evaluated by one or more of three board certified veterinary radiologists (CO, DF, KA) using Carestream Vue PACS (Carestream Health, Rochester, NY) and by a Nuclear Medicine and Diagnostic Radiology board-certified medical radiologist (JF) using Siemens Syngo Via MI Oncology Version 3.0 (VB1) workstation software. All scans were inspected for any areas of increased metabolic activity. Abnormalities, if present, were measured on post-contrast CT scans in the sagittal, transverse, and dorsal planes.

^18^FDG uptake was evaluated subjectively, and a representative region of interest (ROI) was drawn with freehand technique over the maximum size of each lesion on pre- and post-contrast images and mean attenuation was calculated in Hounsfield Units (HU). Maximum standardized uptake value (SUV_max_) and peak SUV (SUV_peak_), defined as the average SUV within a small, fixed-size region of interest (ROI_peak_) centered on a high-uptake portion of suspected tumor lesions as well as other abnormalities were calculated [28]. SUV_max_ and SUV_peak_ of the liver were also calculated for each dog and used as background reference. Contrast enhancement of each lesion was graded as homogeneous, heterogeneous, or ring. Findings from the diagnostic CT portion of the studies were compared to those from the fused PET-CT images.

## Results

The demographic characteristics of the nine dogs that had ^18^FDG-PET-CT scans are summarized in Table 1 and shown for each dog individually in S1 Table. The population was comprised of dogs at risk for this disease, including medium-sized to large (body weight = 30.4 ± 11.7 kg), older dogs (9.2 ± 1.5 years old) from eight breeds, with acceptable to slightly overweight body condition for their size and breed (body condition score = 6.1 ± 1.1). All of the dogs had evidence of hemoabdomen at diagnosis (stage-2). The median time between surgery and performance of the ^18^FDG-PET-CT scan was 18 days (average 21 ± 8 days). A second ^18^FDG-PET-CT scan was performed in three dogs following the detection of a right auricle mass (Dog 2), renal changes (Dog 3), and a liver nodule (Dog 6).

**Table 1:** Summary of demographic characteristics, glucose concentration pre-injection, reference SUV peak and reference SUV max values for all dogs.

(A) PET-CT#1							
All dogs (N =)	Age (years)	Body weight (kg)	^a^BCS	^b^Time from diagnosis to PET-CT (days)	Pre-injection glucose concentration (mg/dl)	^c^SUV peak	SUV Max
9	9.2 ± 1.5	30.4 ± 11.7	6.1 ± 1.1	21 ± 8 Median: 18	79.6 ± 15.0 Median: 82	2.12 ± 0.51 Range: 1.36–2.75	2.23 ± 0.56 Range: 1.44–2.94
(B) PET-CT#2							
3	8.73±1.25	25.23±16.2	5±0	40±4.58 Median: 41	84.3±7.63 Median: 86	2.12 ± 0.51 Range: 1.36–2.75	2.23 ± 0.56 Range: 1.44–2.94

(A) Age (years), body weight (kg), body condition score, median time from diagnosis to PET-CT (days), median pre-injection glucose concentration (mg/dl), and mean reference SUV Peak and Max are illustrated for all nine dogs undergoing the initial PET-CT scan (PET-CT # 1). (B) The same parameters are illustrated for three dogs undergoing a repeat PET-CT scan (PET-CT # 2) on day 21. Values are expressed as Mean±SD.

^a^BCS, body condition score;

^b^PET-CT, positron emission tomography computerized tomography;

^c^SUV, standardized uptake value.

Reference SUV_peak_ and SUV_max_ for ^18^FDG uptake in the liver were calculated for each dog (Table 1). The mean pre-scan glucose concentration for all nine dogs was 79.6 ± 15.0 mg/dL (median = 82 mg/dL). The mean pre-scan glucose concentration for the three dogs where the PET-CT scan was repeated was 84.3 ± 7.63 mg/dL (median = 86 mg/dL).The mean reference SUV_peak_ was 2.12 ± 0.51 (range 1.36 to 2.75) and the mean reference SUV_max_ was 2.23 ± 0.56 (range 1.44 to 2.94). The reference SUV_peak_ and SUV_max_ values were tightly correlated (R^2^ = 0.99), but neither value was correlated with the blood glucose concentrations.

Individual pre-injection glucose concentration values and areas of increased FDG uptake with corresponding SUV_peak_ and SUV_max_ are shown in S2 and S3 Tables. Increased SUVs on the initial PET-CT scans were associated with a benign right humeral bone lesion and with polyarthritis (1 dog), reactive lymph nodes (3 dogs), cortical changes and focal ischemia of the kidney (1 dog), reactivity in the pleural space (1 dog), enhanced muscular activity in the crus of the diaphragm (2 dogs), a tooth root abscess (1 dog), and two lesions with a high index of suspicion for HSA in the right atrium and auricle (1 dog) and in the liver near the apex of the gall bladder (1 dog). The diagnosis of polyarthritis in Dog 1 was supported by joint fluid analysis and culture (left elbow joint) performed 10 days following the PET-CT procedure when the dog presented for biopsy of the right humeral lesion and a history of left forelimb lameness that started the day prior to the biopsy procedure. In this dog, the right prescapular lymph node found to have increased uptake on the PET-CT was also biopsied at that time and was confirmed to be reactive on histopathology.

When analyzing the diagnostic CT portion of the studies, findings included a sclerotic lesion at the right humeral methaphysis with no specific cortical or periosteal reaction or destruction and mild to moderate enhancing regional soft tissues surrounding multiple joints with increased amount of joint fluid in the left elbow joint (Dog 1), a mass associated with the right atrium that was progressive on the second scan (Dog 2), irregular contrast-enhancement along the lateral cortical margin of the left kidney that was no longer observed on the second scan (Dog 3), a focal region of bone lysis surrounding the roots of the left upper incisors (Dog 5), a lesion at the apex of the gall bladder that was progressive on the second scan (Dog 6), and enlarged lymph nodes that corresponded to increased uptake on the PET portion of the studies (Dogs 1–3). The reactivity in the pleural space in Dog 3 and the enhanced muscular activity in the crus of the diaphragm (Dog 4 and 7) noted on the PET studies did not correlate with CT abnormalities.

**Fig 1:**
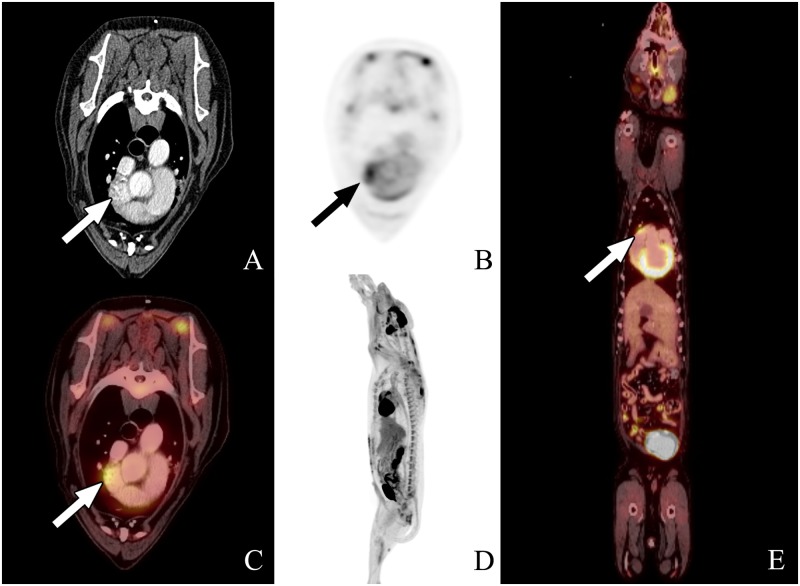
PET-CT scan #1 from dog 2. The images from left to right are transverse (A, B, and C), sagittal (D), and dorsal plane (E), all acquired 1 hour following ^18^FGD injection. A and B represent the CT and PET transverse images, respectively, whereas C is the fused PET-CT transverse image. D represents the whole body ^18^FDG PET-CT scan, and E is the dorsal reconstruction whole body image. Fused and dorsal reconstruction images show increased metabolic activity at the right auricular appendage compared to background tissue. The focal area of uptake at the right auricular appendage is highlighted by the arrow.

**Fig 2:**
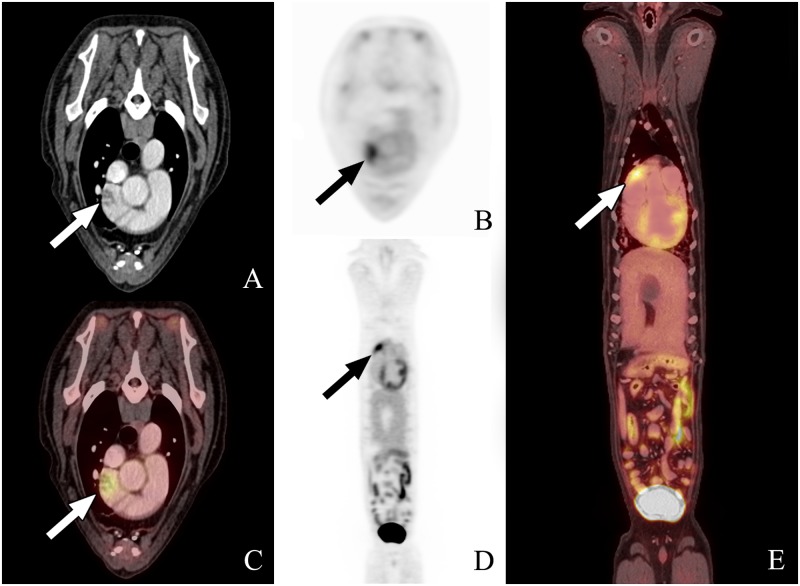
PET-CT scan #2 from dog 2. The images from left to right are transverse (A, B, and C), sagittal (D), and dorsal plane (E), all acquired 1 hour following ^18^FGD injection. A and B represent the CT and PET transverse images, respectively, whereas C is the fused PET-CT transverse image. D represents the whole body ^18^FDG PET-CT scan, and E is the dorsal reconstruction whole body image. Fused and dorsal reconstruction images show further increase in metabolic activity at the right auricular appendage compared to background tissue and growth of the right auricular mass compared to images obtained from PET-CT scan #1. The focal area of uptake at the right auricular appendage is highlighted by the arrow.

**Fig 3:**
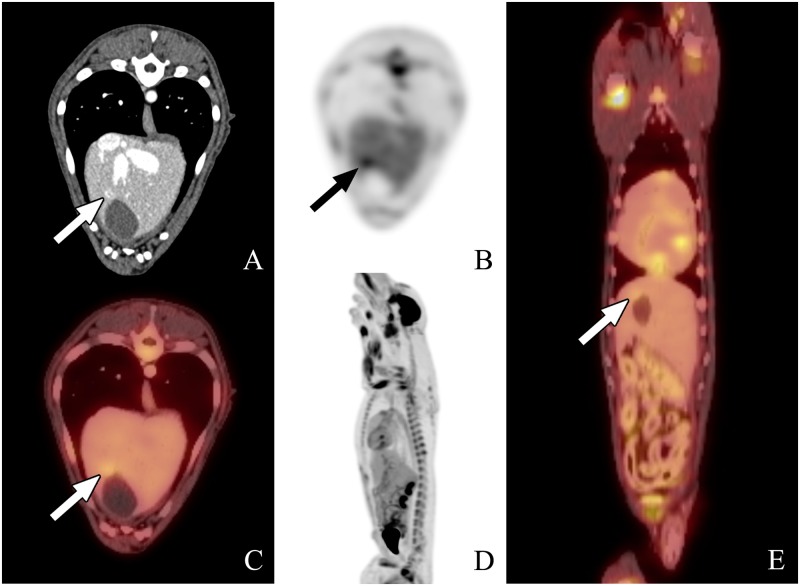
PET-CT scan #1 from dog 6. The images from left to right are transverse (A, B, and C), sagittal (D), and dorsal plane (E), all acquired 1 hour following ^18^FGD injection. A and B represent the CT and PET transverse images, respectively, whereas C is the fused PET-CT transverse image. D represents the whole body ^18^FDG PET-CT scan, and E is the dorsal reconstruction whole body image. There was a focal area of uptake in the liver in proximity of the apex of the gall bladder highlighted by the arrow.

**Fig 4:**
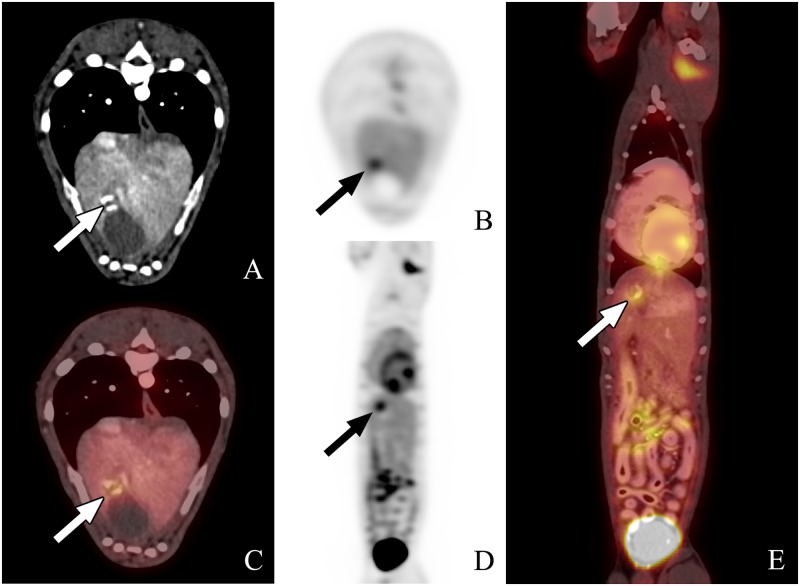
PET-CT scan #2 from dog 6. The images from left to right are transverse (A, B, and C), sagittal (D), and dorsal plane (E), all acquired 1 hour following ^18^FGD injection. A and B represent the CT and PET transverse images, respectively, whereas C is the fused PET-CT transverse image. D represents the whole body ^18^FDG PET-CT scan, and E is the dorsal reconstruction whole body image. The focal area of uptake in the liver in proximity of the apex of the gall bladder is larger and exhibits further increase in metabolic activity compared to PET-CT #1.

The lesions suspected to be consistent with HSA based on PET-CT in two of the nine dogs had not been detected by standard radiography and ultrasonography. The ^18^FDG PET-CT scan in Dog 2 showed increased metabolic activity in the right atrium, auricle, and ventricle. The degree of activity within the left ventricular wall was considered normal whereas the focus of high activity in the right auricular appendage raised suspicion for a malignancy (Fig 1). In this dog, the PET-CT scan was repeated on day 21 and revealed further increase in metabolic activity and size at the right auricular appendage (Fig 2). The ^18^FDG PET-CT scan in Dog 6 showed a focal region of intense radiopharmaceutical activity within the liver near the apex of the gall bladder (Fig 3). This dog also had a PET-CT repeated on day 21 showing increased size and metabolic activity of the hepatic lesion (Fig 4).

Both dogs were eventually euthanatized due to progressive HSA. Dog 2 developed pulmonary nodules radiographically consistent with pulmonary metastatic disease and cytologically-confirmed left renal cortical metastasis 182 days from the diagnosis of HSA. One month later, his referring veterinarian diagnosed congestive heart failure presumably left-sided and secondary to progressive right atrial HSA and euthanasia was performed at the referral institution 223 days from diagnosis. Dog 6 was euthanatized at the referral institution 128 days from diagnosis of HSA due to development of hepatic masses and associated hemoabdomen. A necropsy was not performed in either case. A second PET-CT scan was performed in Dog 3 and previously noted left renal cortical changes, suspected to be associated with focal ischemia, were reevaluated. The renal changes had resolved on the second scan and there was no radiopharmaceutical uptake other than what is expected for normal FDG excretion. Increased uptake was noted in all dogs in kidneys, ureters, and urinary bladder due to normal excretion of FDG. Increased metabolic activity was also noted at the level of cardiac and intestinal muscle, as expected, due to normal activity, and the brain due to avid uptake of glucose by this organ. Most dogs had increased areas of uptake in various skeletal muscles, most likely due to movement and muscular activity prior to the scan.

The PET-CT scans were safe: none of the dogs experienced complications during the procedures. Dogs were kept in isolation until they were dismissed less than 24 hours after the end of the procedure, when radioactive surface emissions measured ≤ 0.5 mR/hr at 1-meter.

## Discussion

To our knowledge, this is the first study to evaluate the feasibility and potential use of PET-CT in dogs with HSA. Our data show that ^18^FDG-PET-CT can be safely performed in dogs following splenectomy, and that this modality can be used to detect potential HSA lesions that are otherwise not identifiable using conventional radiographs and/or ultrasound.

The right atrial mass and the hepatic nodule identified in dogs in this study, and that were presumed to be HSA, were not evaluated cytologically or histopathologically due to their location and the potential risks associated with sampling. They were suspected to represent manifestations of malignant disease based on their growth, appearance, location, increased uptake and metabolic activity on repeat PET-CTs, and the final outcome of progressive HSA in both dogs. A previous study evaluating ^18^FDG uptake in nine canine mesenchymal tumors reported mean standard uptake values between 1.2 and 5.8, and maximum standard uptake values between 2.0 and 10.6, which is consistent with our findings, although none of the cases in this study represented HSAs [6]. Nevertheless, future studies will be required to determine if active bleeding, inflammation or resolving hematomas enhance ^18^FDG uptake in HSA, especially if this modality is intended to monitor response to non-surgical therapies or minimal residual disease.

Currently, there are no standardized imaging guidelines that help distinguish between benign and malignant cardiac tumors in dogs. For this reason, while echocardiography is utilized as a staging tool by some, it is not routine practice at our institution for staging of canine HSA. In the literature, only few isolated reports have described the use of PET-CT in human patients with primary cardiac masses to assess their malignant potential and to provide preoperative staging information and monitor response to treatment [29–32]. It is possible that an SUV cut off for benign versus malignant lesions could be identified in dogs with different histologies. Further evaluation of PET-CT imaging in dogs could provide important cues in the diagnosis of cardiac masses before treatment recommendations are made.

Metastatic dissemination of HSA occurs mainly by the hematogenous pathway. Common sites of involvement include lungs and liver, but other sites, such as bone and central nervous system (CNS) can also be affected in some cases. While pulmonary metastatic disease can be frequently detected by conventional radiography, and abdominal metastases are commonly identified by abdominal ultrasonography, skeletal radiographs or bone scintigraphy to detect bone metastasis and magnetic resonance imaging (MRI) to identify CNS lesions are not routinely conducted before or after splenectomy is performed or the decision to pursue chemotherapy is made by the pet’s owners. Notably, a study comparing ^18^FDG-PET-CT scans and technetium-99m methylene diphosphonate (99Tc-MDP) bone scans in 29 pediatric patients with bone and soft tissue sarcomas showed that PET-CT was able to detect all malignant bone lesions compared to a 99Tc-MDP accuracy of 80%, leading to the conclusion that ^18^FDG-PET-CT was equivalent or superior as a diagnostic modality [33]. In our study, PET-CT identified a bone lesion on the right proximal humerus of one dog. Based on appearance and SUVs, this lesion was suspected to be benign in nature. Histopathology confirmed sclerotic changes with no evidence of a malignancy. The dog was euthanatized at the referral institution due to progressive HSA 484 days from diagnosis.

Because the nature of a splenic mass is often unknown prior to surgery and because the identification of widespread lesions could profoundly influence the decision to proceed with treatment, improved imaging modalities that provide more accurate and complete staging information represent an unmet need in veterinary medicine. ^18^FDG-PET-CT could fill this gap. Even though widespread adoption of this modality will require greater availability and significant cost reductions, these barriers have been largely overcome by other advanced imaging methods, such as ultrasound, computerized tomography, and magnetic resonance imaging over the past two decades. Unlike the more commonly utilized procedure to incorporate low resolution CT studies to PET studies, our ^18^FDG-PET-CTs consisted of diagnostic quality CT scans that allowed the identification of abnormalities that, for the most part, correlated with the increased uptake on PET. Therefore, it is arguable that CT scans alone could sufficiently improve the diagnostic quality of current staging and monitoring protocols. While this may be true, our data indicate that ^18^FDG-PET-CT has potential to complement and expand the quality and quantity of information that can be obtained from these currently accepted technologies, and thus help improve the management of veterinary cancer patients. It must be acknowledged that the interpretation of PET-CT studies presents several challenges. One of the main challenges is the variability in FDG uptake by normal tissues. Other factors include FDG uptake related to inflammation or infection, malignant lesions with unusually low avidity for FDG, altered biodistribution of FDG related to hyperglycemia, and bone marrow activation, among others [34]. While motion artifacts are of greater concern in people, they are minimized in veterinary practice by the requirement of general anesthesia for PET-CT scanning. These considerations are not only important to the interpreting radiologist, but also to the attending veterinarian responsible for assessing the clinical significance of the findings and making further diagnostic recommendations. These pitfalls will be likely reduced as our knowledge of how metabolic activity varies in benign and malignant lesions expands and SUV cut-offs are identified to aid the diagnosis of disease processes through minimally invasive approaches.

In conclusion, PET-CT is a promising tool for staging and monitoring of canine HSA and studies using larger populations are warranted to further evaluate the sensitivity of this imaging modality for staging, to assess therapeutic responses, and to monitor duration of remission. Ideally, necropsy should be a requirement moving forward to correlate the PET-CT characteristics of any possible lesions with histopathology. These studies will be crucial as the availability of PET-CT increases at veterinary institutions and novel targeted therapies are developed.

## Supporting information

S1 Table Demographic characteristics of dogs in the study. Age (years), sex and neuter status, body condition score, presence of hemoabdomen, stage, and time (days) from diagnosis to PET-CT#1 and PET-CT#2 (where applicable) for the nine dogs enrolled in the study are shown.
(DOCX)

S2 Table PET-CT # 1 findings with mean and max SUVs. Areas of increased FDG uptake on the initial PET-CT scans (PET-CT #1), their presumed diagnosis based on appearance and SUV peak and max are shown for the nine dogs enrolled in the study and the pre-injection glucose concentration values (mg/dl) are listed for each dog.
(DOCX)

S3 Table PET-CT # 2 findings with mean and max SUVs. Areas of increased FDG uptake on the repeat PET-CT scans (PET-CT #2), their presumed diagnosis based on appearance and SUV peak and max are shown for the three dogs in which scans were repeated, and the pre-injection glucose concentration values (mg/dl) are listed for each dog.
(DOCX)
