# Supplementary material for: Evaluation of 18-F-fluoro-2-deoxyglucose (FDG) positron emission tomography/computed tomography (PET/CT) as a staging and monitoring tool for dogs with stage-2 splenic hemangiosarcoma – A pilot study
Source: PLoS One. 2017 Feb 21;12(2):e0172651. doi: 10.1371/journal.pone.0172651 (PMC5319762; doi:10.1371/journal.pone.0172651)
Supplement: S1 Table — Age (years), sex and neuter status, body condition score, presence of hemoabdomen, stage, and time (days) from diagnosis to PET-CT#1 and PET-CT#2 (where applicable) for the nine dogs enrolled in the study are shown. (DOCX) [file pone.0172651.s001.docx]

**S1 Table. Demographic Characteristics of Dogs in the Study**

| Dog  1 | Age  (Years)  11.5 | Sex and neuter status  MN | Breed  Airedale Terrier | Weight  (kg)  28.9 | BCS  5 | Hemoabdomen  Y | Stage  2 | Time from diagnosis to PET-CT  (days)  #1 #2  25 |  |
| --- | --- | --- | --- | --- | --- | --- | --- | --- | --- |
| 2 | 10.3 | MN | Newfoundland | 44.2 | 6 | Y | 2 | 15 35 |  |
| 3 | 8.5 | FS | Viszla | 21.6 | 6 | Y | 2 | 18 41 |  |
| 4 | 10.0 | FS | Goldendoodle | 32.0 | 7 | Y | 2 | 17 |  |
| 5 | 7.1 | FS | Labrador Retriever | 41 | 8 | Y | 2 | 39 |  |
| 6 | 7.4 | MN | Dachshund | 9.3 | 5 | Y | 2 | 23 44 |  |
| 7 | 8.1 | MN | German Shepherd | 43.7 | 6 | Y | 2 | 22 |  |
| 8 | 10.2 | MN | Labrador Retriever | 21.5 | 5 | Y | 2 | 14 |  |
| 9 | 9.8 | MN | Golden Retriever | 31.8 | 7 | Y | 2 | 15 |  |

*BCS, body condition score; PET-CT, positron emission tomography computerized tomography
