# Supplementary material for: Evaluation of 18-F-fluoro-2-deoxyglucose (FDG) positron emission tomography/computed tomography (PET/CT) as a staging and monitoring tool for dogs with stage-2 splenic hemangiosarcoma – A pilot study
Source: PLoS One. 2017 Feb 21;12(2):e0172651. doi: 10.1371/journal.pone.0172651 (PMC5319762; doi:10.1371/journal.pone.0172651)
Supplement: S2 Table — Areas of increased FDG uptake on the initial PET-CT scans (PET-CT #1), their presumed diagnosis based on appearance and SUV peak and max are shown for the nine dogs enrolled in the study and the pre-injection glucose concentration values (mg/dl) are listed for each dog. (DOCX) [file pone.0172651.s002.docx]

**S2 Table. PET-CT # 1 Findings with Mean and Max SUVs**

| Dog | Pre-injection glucose concentration (mg/dl) | Areas of increased FDG uptake | Presumed diagnosis | SUV Peak | SUV Max |
| --- | --- | --- | --- | --- | --- |
| 1  2 | 80  82 | Liver  Proximal  methaphysis of  right humerus  Left elbow joint  Left scapulo-  humeral joint  Coxo-femoral  joints  Liver  Right atrium, auricle and ventricles  Left submandibular lymph node | Reference/background  Benign bone lesion  Polyarthritis  Polyarthritis  Polyarthritis  Reference/background  Suspected auricular hemangiosarcoma  Reactive | 2.61  2.84  3.72  2.84  2.33  2.75  5.47  4.02 | 2.68  3.49  4.86  3.49  2.80  2.94  6.61  4.6 |
| 3 | 101 | Liver  Normal kidney  Left kidney  Pleural space  Portal lymph node | Reference/background  Reference/background  Likely ischemia  Reactive  Reactive | 2.03  2.25  5.34  2.48  2.45 | 2.15  2.17  6.31  1.91  3.02 |
| 4 | 98 | Liver  Crus of diaphragm | Reference/background  Muscle activity | 1.91  7.6 | 2.01  5.65 |
| 5 | 68 | Liver  Left upper incisors | Reference/background  Tooth root abscess | 2.70  3.70 | 2.94  6.05 |
| 6 | 57 | Liver  Liver near the apex of the gall bladder | Reference/background  Suspected hemangioma versus early hemangiosarcoma metastasis | 1.72  2.06 | 1.81  2.33 |
| 7 | 70 | Liver  Crus of diaphragm | Reference/background | 1.36  4.65 | 1.44  6.21 |
| 8 | 67 | Liver  No abnormal areas of uptake noted | Reference/background | 2.38 | 2.51 |
| 9 | 84 | Liver  No abnormal areas of uptake noted | Reference/background | 1.64 | 1.63 |

*PET-CT, positron emission tomography computerized tomography; SUV, standardized uptake value; FDG, fluoro-deoxy-glucose.
