# Supplementary material for: Evaluation of 18-F-fluoro-2-deoxyglucose (FDG) positron emission tomography/computed tomography (PET/CT) as a staging and monitoring tool for dogs with stage-2 splenic hemangiosarcoma – A pilot study
Source: PLoS One. 2017 Feb 21;12(2):e0172651. doi: 10.1371/journal.pone.0172651 (PMC5319762; doi:10.1371/journal.pone.0172651)
Supplement: S3 Table — Areas of increased FDG uptake on the repeat PET-CT scans (PET-CT #2), their presumed diagnosis based on appearance and SUV peak and max are shown for the three dogs in which scans were repeated, and the pre-injection glucose concentration values (mg/dl) are listed for each dog. (DOCX) [file pone.0172651.s003.docx]

**S3 Table. PET-CT # 2 Findings with Mean and Max SUVs**

| Dog | Pre-injection glucose concentration (mg/dl) | Areas of increased FDG uptake | Presumed diagnosis | SUV Peak | SUV Max |
| --- | --- | --- | --- | --- | --- |
| 2 | 76 | Liver  Right atrium, auricle and ventricles  Left submandibular lymph node | Reference/background  Suspected auricular hemangiosarcoma  Reactive | 2.75  6.9  2.14 | 2.94  7.95  2.58 |
| 3 | 86 | Liver  Normal kidney  Left kidney  Portal lymph node | Reference/background  Reference/background  Normal  Reactive | 2.03  2.25  2.9  3.01 | 2.15  2.17  2.88  3.72 |
| 6 | 91 | Liver  Liver near the apex of the gall bladder | Reference/background  Suspected hemangioma versus early hemangiosarcoma metastasis | 1.72  2.97 | 1.81  3.97 |

*PET-CT, positron emission tomography computerized tomography; SUV, standardized uptake value; FDG, fluoro-deoxy-glucose.
